# Genetic Ablation of PLA2G6 in Mice Leads to Cerebellar Atrophy Characterized by Purkinje Cell Loss and Glial Cell Activation

**DOI:** 10.1371/journal.pone.0026991

**Published:** 2011-10-28

**Authors:** Zhengshan Zhao, Jing Wang, Chunying Zhao, Weina Bi, Zhenyu Yue, Zhongmin Alex Ma

**Affiliations:** 1 Division of Experimental Diabetes and Aging, Department of Geriatrics and Palliative Medicine, Mount Sinai School of Medicine, New York, New York, United States of America; 2 Department of Neurology, Mount Sinai School of Medicine, New York, New York, United States of America; Medical College of Georgia, United States of America

## Abstract

Infantile neuroaxonal dystrophy (INAD) is a progressive, autosomal recessive neurodegenerative disease characterized by axonal dystrophy, abnormal iron deposition and cerebellar atrophy. This disease was recently mapped to *PLA2G6*, which encodes group VI Ca^2+^-independent phospholipase A_2_ (iPLA_2_ or iPLA_2_β). Here we show that genetic ablation of PLA2G6 in mice (iPLA_2_β^-/-^) leads to the development of cerebellar atrophy by the age of 13 months. Atrophied cerebella exhibited significant loss of Purkinje cells, as well as reactive astrogliosis, the activation of microglial cells, and the pronounced up-regulation of the pro-inflammatory cytokines tumor necrosis factor-α (TNF-α) and interleukin-1β (IL-1β). Moreover, glial cell activation and the elevation in TNF-α and IL-1β expression occurred before apparent cerebellar atrophy. Our findings indicate that the absence of PLA2G6 causes neuroinflammation and Purkinje cell loss and ultimately leads to cerebellar atrophy. Our study suggests that iPLA_2_β^-/-^ mice are a valuable model for cerebellar atrophy in INAD and that early anti-inflammatory therapy may help slow the progression of cerebellar atrophy in this deadly neurodegenerative disease.

## Introduction

The phospholipase A_2_ (PLA_2_) family is a diverse group of enzymes that catalyzes the hydrolysis of the *sn*-2 fatty acyl bond of phospholipids to liberate free fatty acids and lysophospholipids [1]. Among the family members, group VIA PLA_2_ (iPLA_2_β) has unique structural features, including eight N-terminal ankyrin repeats, caspase-3 cleavage sites, an ATP-binding domain, a serine lipase consensus sequence (GXSXG), a bipartite nuclear localization sequence, and a C-terminal calmodulin-binding domain [1]. The human iPLA_2_β gene, *PLA2G6*, maps to chromosome 22q13.1 and encodes several isoforms [2].

Morgan et al. [3] reported that mutations in *PLA2G6* underlie human neurodegenerative disorders, infantile neuroaxonal dystrophy (INAD) and neurodegeneration with brain iron accumulation (NBIA). INAD is a neurodegenerative disease with infantile onset and death as a teenager or in early adulthood. It is characterized by pathologic axonal swelling and spheroid bodies in the peripheral and central nervous systems (CNS) [4], [5], [6]. Mutations in PLA2G6 have recently been identified in many INAD patients, suggesting that iPLA_2_β plays an essential role in the CNS and the development of the disorder.

We used homologous recombination to generate iPLA_2_β-knockout mice (iPLA_2_β^-/-^) [7]. These mice developed severe motor dysfunction associated with the prominent formation of spheroids with tubulovesicular membranes remarkably similar to those seen in human INAD [8], [9]. Consistent with the recessive nature of INAD, mutagenesis analysis demonstrated that the catalytic function of the INAD-associated mutated PLA2G6 is impaired [10]. Thus, iPLA_2_β^-/-^ mouse is a useful animal model of the pathogenesis of INAD.

The neuroradiological hallmark of INAD is the cerebellar atrophy. Marked cerebellar atrophy is the most characteristic MRI feature of INAD patients [11], [12], [13], [14] and observed in almost all INAD patients with mutations in *PLA2G6* [3], [4], [5], [6], [15], [16], [17], [18], suggesting a neuropathologic role of dysfunctional iPLA_2_β in the cerebellar atrophy in the INAD patients. Here, we report that iPLA_2_β^-/-^ mice develop severe cerebellar atrophy — characterized by the loss of Purkinje neurons, the activation of glial cells, and the elevation of pro-inflammation cytokines — by the age of 13 months. Our findings provide insight into the pathological mechanisms of cerebellar atrophy in human INAD.

## Results

### Cerebellar atrophy and Purkinje cell loss in iPLA_2_β^-/-^ mice

**Figure 1:**
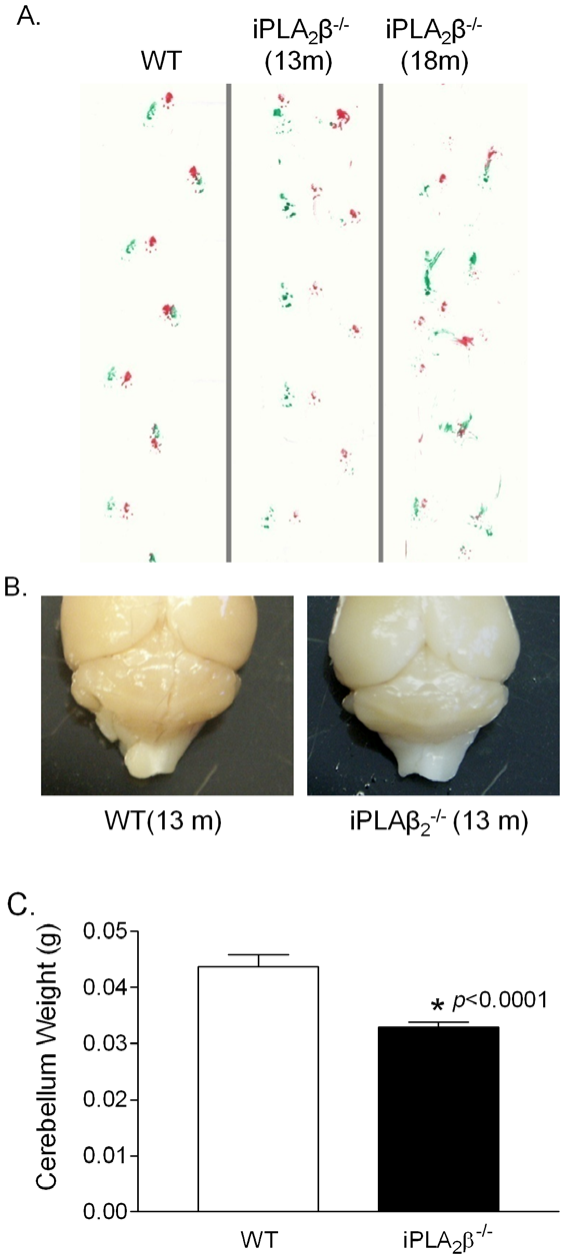
Ataxia and cerebellar atrophy in the iPLA_2_β^-/-^ mouse. A. Representative footprint patterns in gait stride tests of WT and iPLA2β^-/-^ mice. B. Representative cerebellum of iPLA2β^-/-^ mouse. C. Cerebella from the iPLA2β^-/-^ mice (13–18 months) and the age-matched WT mice (n = 15/group) were weighted and statistically analyzed.* p<0.0001.

Cerebellar ataxia is an early symptom of INAD patients with PLA2G6 mutations [6]. We found that iPLA_2_β^-/-^ mice exhibited ataxia and irregular strides by the age of 13 months, and that these conditions became more severe with age (Fig 1A). Furthermore, the cerebella of the iPLA_2_β^-/-^ mice were noticeably smaller than that of the age-matched WT littermates (Fig. 1B) and they weighed nearly 25 percent less (Fig 1C). These findings indicated that iPLA_2_β^-/-^ mice develop cerebellar atrophy by the age of 13 months that resembles the atrophy seen in humans with INAD.

**Figure 2:**
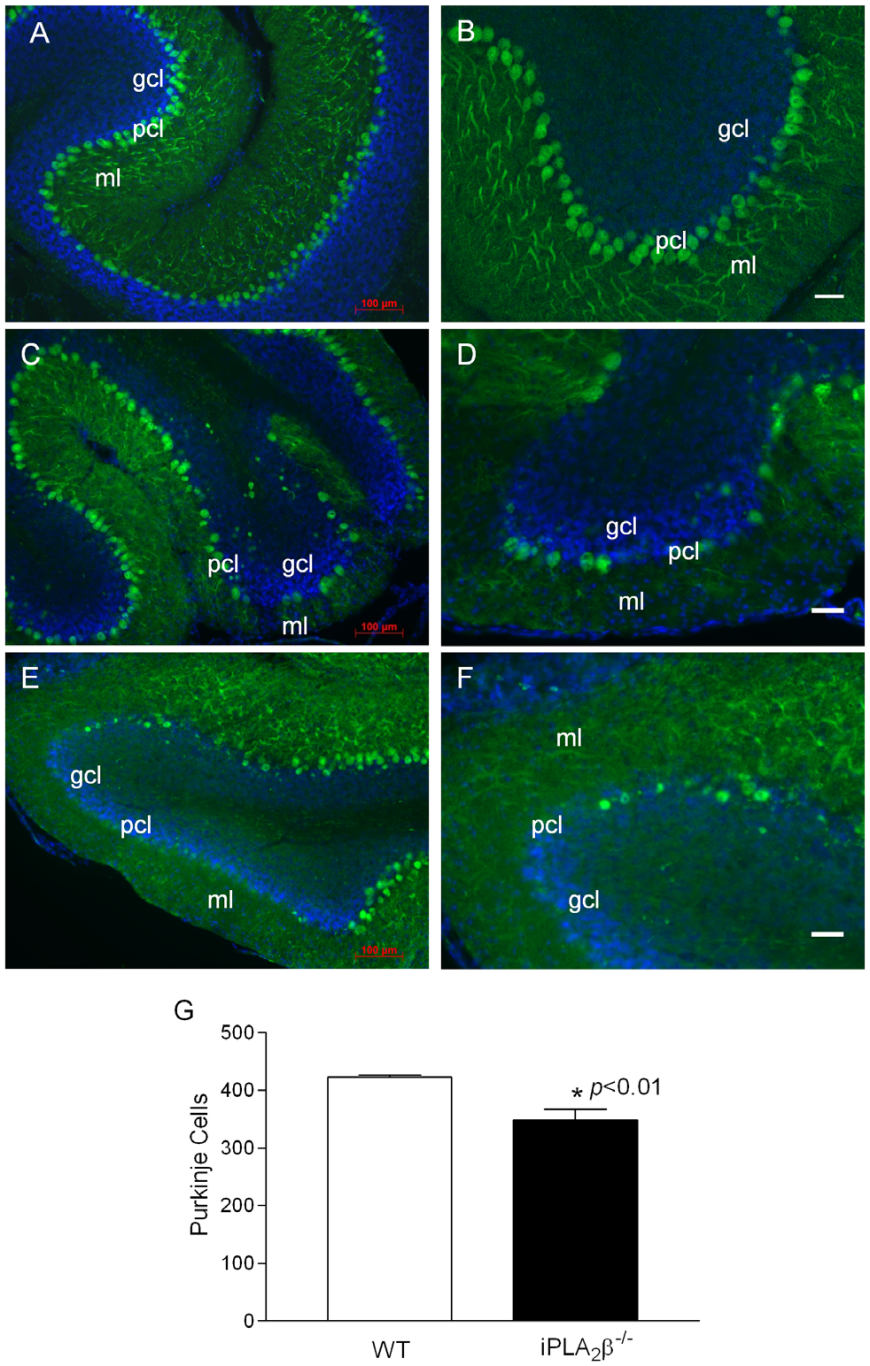
Purkinje cell loss in the iPLA2β^-/-^ mouse. A, B. Representative calbindin staining in the cerebella of the WT mice (18 months). C, D. Representative calbindin staining in the cerebella of the iPLA_2_β^-/-^ mice (13 months), and E, F. (18 months). G. Purkinje cell counting in WT (n = 6) and iPLA_2_β^-/-^ mice (n = 8) at the age of 13–18 months. p<0.01. Blue color, DAPI staining; pcl, Purkinje cell layer; ml, molecular layer; gcl, granule cell layer; wm, white matter. White scale bar, 50 µm.

We next analyzed cerebellar sections from the iPLA_2_β^-/-^ mice to determine the mechanism of cerebellar atrophy. In agreement with a previous report [8], parasagittal views of nissl-stained mouse cerebella revealed no apparent anatomical differences between the iPLA_2_β^-/-^ mice and their WT littermates (Figure S1). However, when we examined the expression of calbindin, a widely used immunohistochemical marker of Purkinje neurons in the cerebellum [19], we found that, in comparison to the WT controls (Fig. 2A–B), the Purkinje cell layer and the molecular layer in the iPLA_2_β^-/-^ mice are disrupted (Fig. 2C–F). By stereological counting of calbindin-positive cells (Figure S2), we found that iPLA_2_β^-/-^ mice have significantly fewer Purkinje cells that the WT controls (Fig. 2J). Our data suggest that Purkinje cell loss is a pathologic characteristic of cerebellar atrophy in iPLA_2_β^-/-^ mice.

### Glial cell activation in the cerebella of iPLA_2_β^-/-^ mice

**Figure 3:**
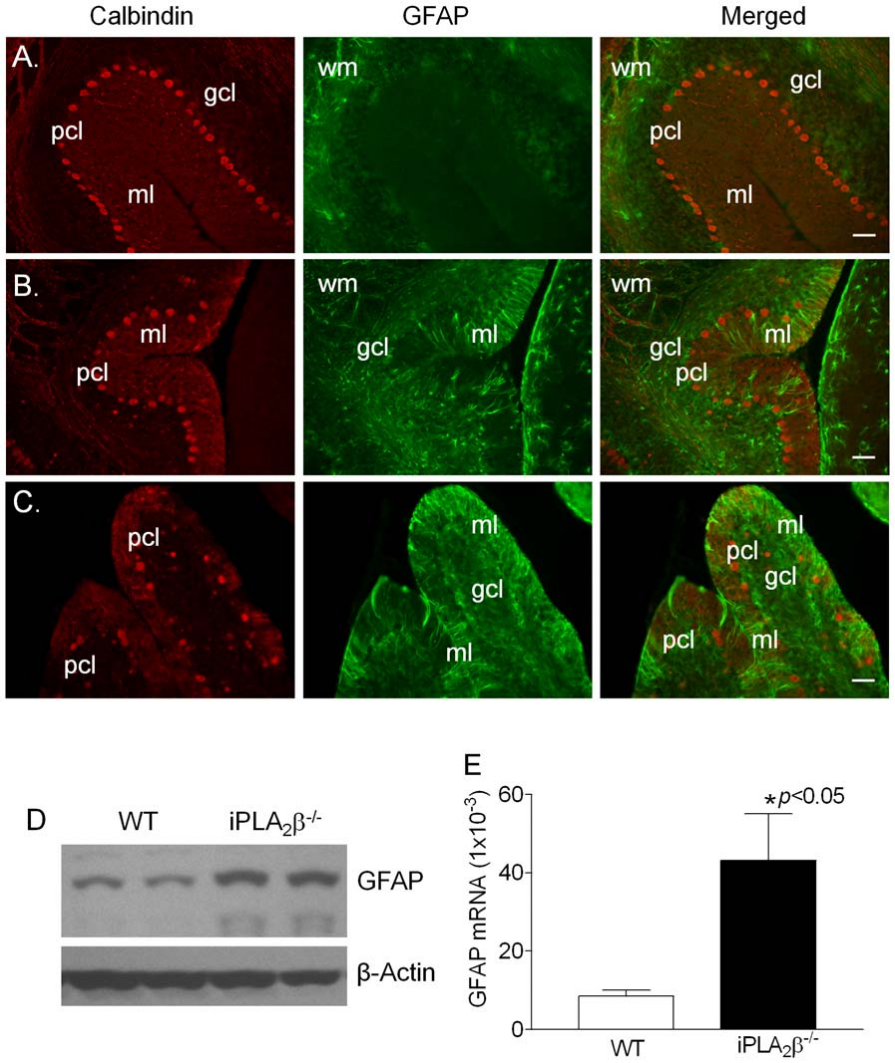
Immunofluorescent analyses of GFAP in the cerebella. (A) Representative GFAP/calbindin double-staining in the cerebella of the WT mice at the age of 13–18 months (n = 12). (B, C) GFAP/calbindin double-staining in the cerebella of the iPLA_2_β^-/-^ mice at the age of 13–18 months (n = 14). D. Western blot analysis of GFAP in the cerebellum of iPLA_2_β^-/-^. E. qRT-PCR analysis of the cerebellar GFAP mRNA (n = 6 each). pcl, Purkinje cell layer; ml, molecular layer; gcl, granule cell layer; wm, white matter. Scale bar, 50 µm.

Cerebellar atrophy observed in INAD patients is often associated with cerebellar gliosis, which is characterized by signal hyperintensity in the cerebellar cortex on T2-wighted images of MRI [6]. Since reactive astrogliosis has long been recognized as a ubiquitous feature of CNS pathologies [20], we determined whether reactive astrogliosis occurs in the cerebella of the iPLA_2_β^-/-^ mice by examining the expression of glial fibrillary acidic protein (GFAP), of which up-regulation is the best known hallmark of reactive astrogliosis in CNS [20]. We found that, with the exception of the white matter, GFAP levels were very low in the cerebella of the WT mice (Fig. 3A). However, GFAP levels were markedly increased in the molecular and granule cell layers of the cerebella of the iPLA_2_β^-/-^ mice (Fig. 3B and 3C). In consistent with the immunostaining, Western blot analysis (Fig 3D) and real time quantitative RT-PCR (Fig. 3E) also showed the significant increase in GFAP expression in the cerebella of the iPLA_2_β^-/-^ mice. This pronounced increase of GFAP in the molecular and granule cell layers of the cerebella of the iPLA_2_β^-/-^ mice indicate that reactive astrogliosis is a characteristic feature of cerebellar atrophy in iPLA_2_β^-/-^ mice.

**Figure 4:**
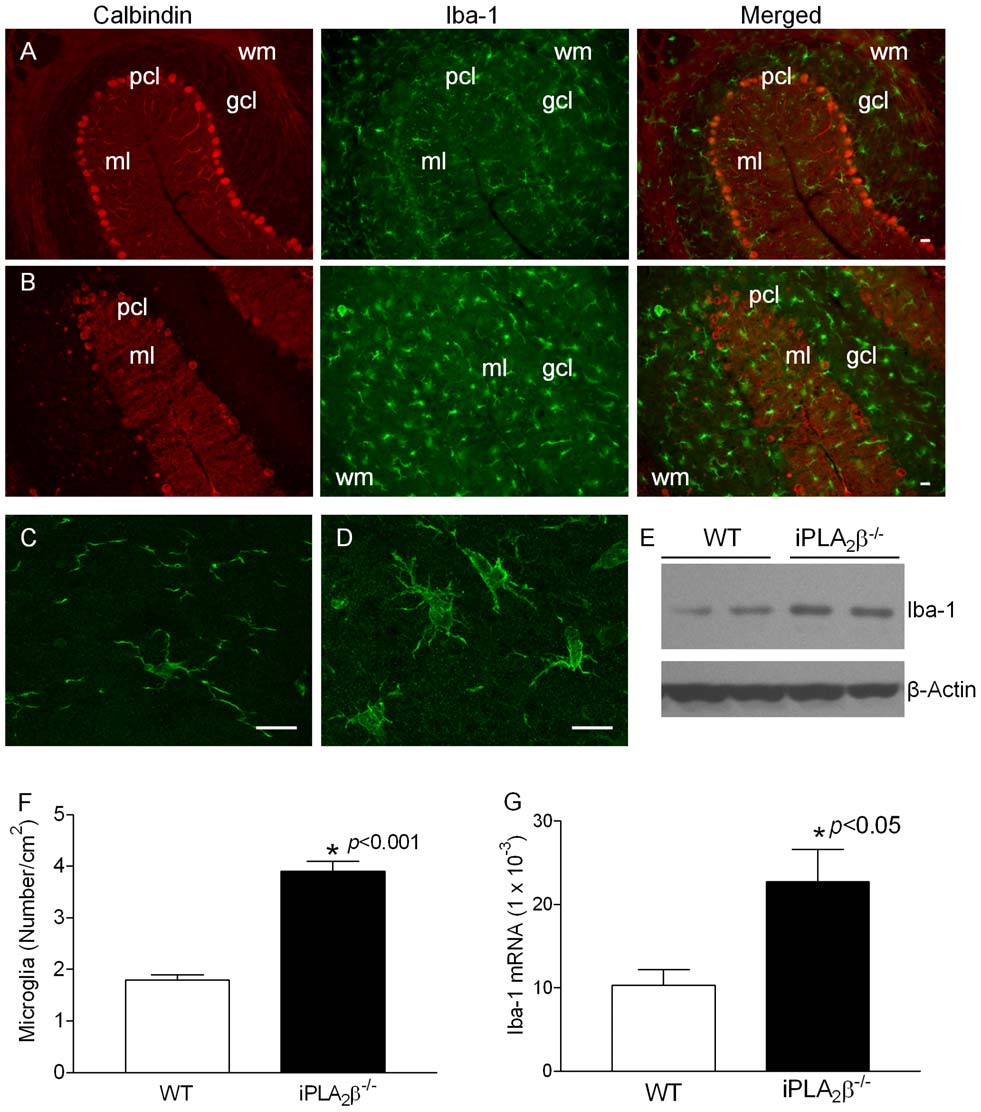
Immunofluorescent analyses of Iba-1 in the cerebella. A. Representative Iba-1/calbindin double-staining in the cerebella of the 13–18 month-old WT mice. B. Representative Iba-1/calbindin double-staining in the cerebella of the iPLA_2_β^-/-^ mice at the age of 13–18 months. Confocal microscopy analysis revealed ramified microglia with small cellular bodies and thin branches in the cerebella of the WT (C) and activated microglia with amoeboid morphology, larger cellular bodies, and thick branches in the cerebella of the iPLA_2_β^-/-^ mice (D). E. Western blot analysis of GFAP in the cerebellum of iPLA_2_β^-/-^. F. Quantitation of microglial cells in the molecular layer of the mouse cerebellum at the age of 13–18 months. G. qRT-PCR analysis of the cerebellar Iba-1 mRNA (n = 6 each) at the age of 13–18 months. ml, molecular layer; gcl, granule cell layer. Scale bar, 20 µm.

Microglial activation plays a central role in reactive astrogliosis [21]. To determine whether microglial activation also occurs in the cerebella in the iPLA_2_β^-/-^ mice, we examined the expression of ionized calcium binding adaptor molecule 1 (Iba-1) – a 17-kDa EF hand protein that is specifically expressed in brain microglia and is up-regulated during their activation [22]. Iba-1 immunostaining revealed ramified microglia with small cellular bodies in the molecular layer and granule cell layer (Fig. 4A, 4C) of the WT cerebella. In contrast, microglia in the molecular layer and granule cell layer of the iPLA_2_β^-/-^ cerebella displayed features of activation, including an amoeboid morphology with larger cellular bodies, thick branches (Fig. 4B, 4D), increased cell numbers (Fig. 4F), and up-regulated of Iba-1 expression (Fig. 4E, G) [23].

### Elevation of proinflammatory cytokines

**Figure 5:**
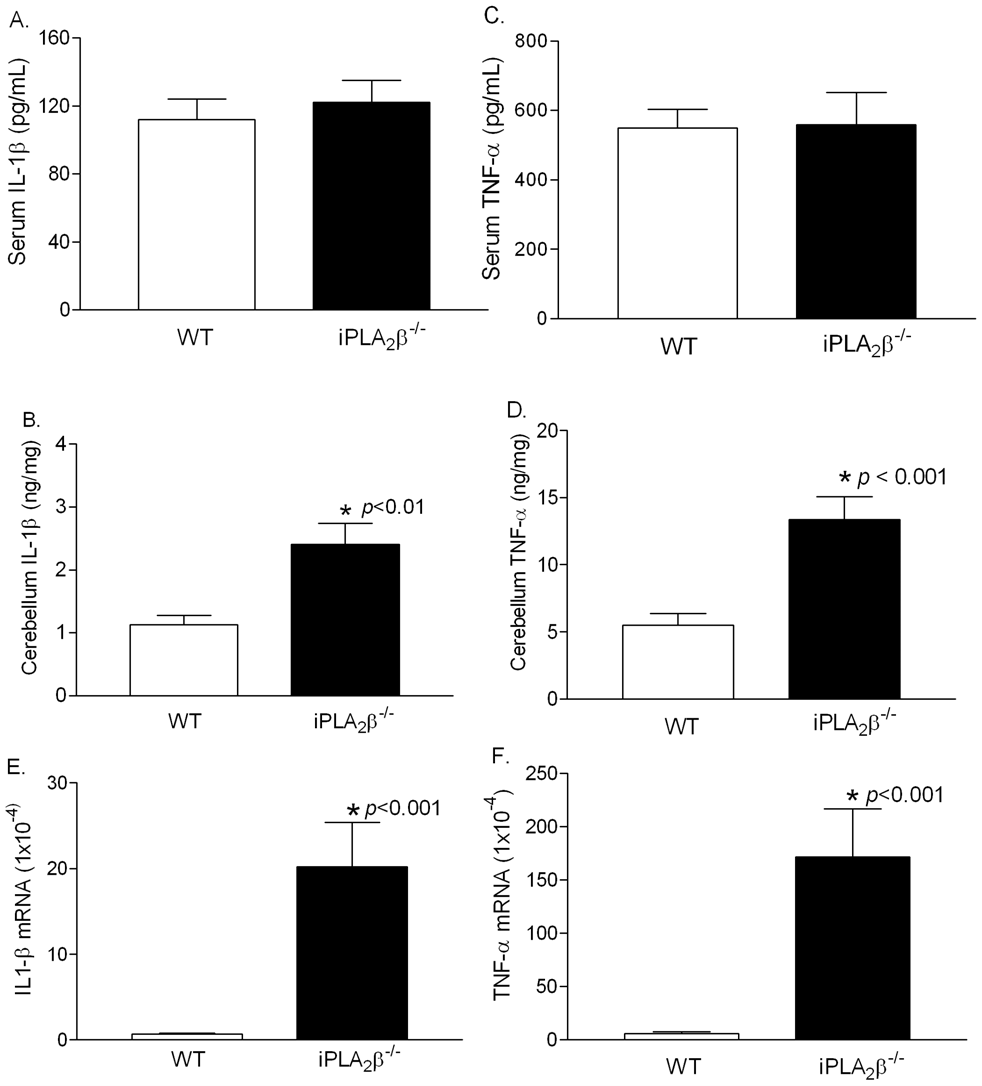
Upregulation of IL-1β and TNF-α in the cerebella of the iPLA_2_β^-/-^ mice. A. Serum IL-1β levels (WT, n = 8; iPLA_2_β^-/-^, n = 10). B. Serum TNF-α levels (WT, n = 8; iPLA_2_β^-/-^, n = 10). C. Cerebella IL-1β levels (WT, n = 6; iPLA_2_β^-/-^, n = 6). D. Cerebella TNF-α levels (WT, n = 6; iPLA_2_β^-/-^, n = 6). E. Cerebella IL-1β mRNA levels (WT, n = 3; iPLA_2_β^-/-^, n = 3). F. Cerebella TNF-α mRNA levels (WT, n = 3; iPLA_2_β^-/-^, n = 3).

Activation of microglia often results in the release of pro-inflammatory cytokines such as TNF-α and IL-1β [24], [25]. We found that although the levels of TNF-α and IL-1β in the serum of iPLA_2_β^-/-^ and WT mice do not differ (Fig. 5A–B), the cerebella of iPLA_2_^-/-^ mice expressed significantly higher levels of these cytokines (Fig. 5C–D) and their mRNA transcripts (Fig. 5E–F). Our findings demonstrate that Purkinje cell degeneration is tightly associated with inflammation in the cerebella of the iPLA_2_β^-/-^ mice.

### Glial cell activation and TNF-α and IL-1β production without apparent cerebellar atrophy

**Figure 6:**
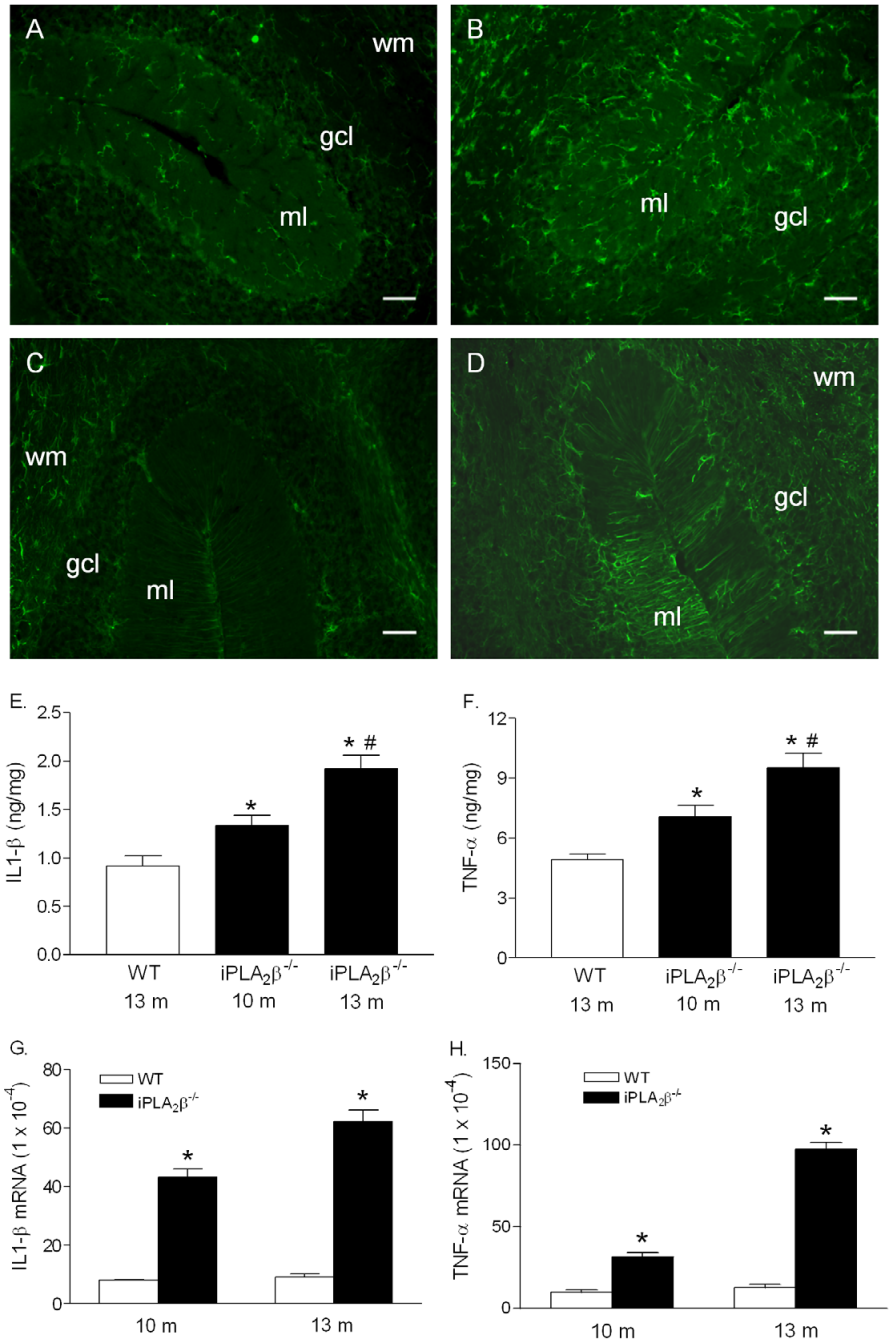
Glial cell activation and elevation of IL-1β and TNF-α in the cerebella of the iPLA_2_β^-/-^ mice before apparent cerebellar atrophy. A. Representative Iba-1 staining in the cerebella of the WT mice (10-month, n = 3) and (B) in the cerebella of the iPLA_2_β^-/-^ mice (10-month, n = 6). C. Representative GFAP staining in the cerebella of the WT mice (10-month, n = 3) and (D) in the cerebella of the iPLA_2_β^-/-^ mice (10-month, n = 6). Scale bar, 100 µm. E. Cerebella IL-1β levels (n  =  4 in each group). F. Cerebellar TNF-α levels (n = 4 in each group). G. Cerebella IL-1β mRNA levels (n = 3 in each group). H. Cerebella TNF-α mRNA levels (n = 3 in each group). * vs. the WT, p<0.01; #vs. the 10-month iPLA_2_β^-/-^ mice, p<0.01.

Cerebellar atrophy in the iPLA_2_β^-/-^ mice does not become apparent until the age of 13 months. However, we found that microglia (Fig. 6B) and astrocytes (Fig. 6D) are activated in the cerebella of iPLA_2_β^-/-^ mice as young as 10 months (Fig. 6A, 6C). These mice also exhibit significantly increased levels of IL-1β (Fig. 6E) and TNF-α (Fig. 6F) in their cerebella, which further increase with age. These elevated cytokine levels can be attributed to local protein production, as demonstrated by elevated mRNA levels of IL-1β (Fig. 6G) and TNF-α (Fig. 6H) in the iPLA_2_β^-/-^ mice. Since we observed no significantly difference in the Purkinje cell layer between the WT and iPLA_2_β^-/-^ mice at this age, our findings suggest that inflammation in the cerebella of iPLA_2_β^-/-^ mice may be one contributing factor leading to Purkinje cell loss and subsequent cerebellar atrophy.

## Discussion

In this study, we used a mouse model of INAD [8], [9] to investigate potential mechanisms of cerebellar atrophy — a hallmark of INAD patients with *PLA2G6* mutations [3], [4], [5], [6], [15], [16], [17], [18]. We found that, by the age of 13 months, iPLA_2_β^-/-^ mice exhibited a loss of Purkinje cells and their dendrites in the molecular layer of the cerebella. Our results indicate that Purkinje cell loss is a pathologic characteristic of cerebellar atrophy in iPLA_2_β^-/-^ mice.

An important finding in our study is that the cerebellar atrophy observed in iPLA_2_β^-/-^ mice is associated with reactive astrogliosis, overactivation of microglial cells, and pronounced upregulation of pro-inflammatory cytokines including IL-1β and TNFα — all features of neuroinflammation associated with a vast array of CNS pathologies [26]. Importantly, these features were observed not only in the cerebella of 13-month-old iPLA_2_β^-/-^ mice with apparent neurological abnormality, but also in younger mice prior to the onset of overt disease. Our findings suggest that inflammation may play a pathological role in the development of cerebellar atrophy in INAD.

Microglia typically exist in a resting state characterized by ramified morphology (Fig. 3C) monitoring the brain environment and they are usually the first cells to be activated by pro-inflammatory stimuli, such as lipopolysaccharide (LPS), or in response to neuronal damage [24]. In their activated state, they can serve diverse beneficial functions essential to neuron survival including cellular maintenance, innate immunity, and regulating brain development by enforcing the programmed elimination of neural cells [24]. Indeed, activation of microglia has been reported to promote the death of developing Purkinje cells during brain development [27]. However, under certain circumstances, microglia become overactivated and release excess TNF-α and IL-1β, which further activate astrocytes and spark a perpetuating cycle of neuron death [21].

What could cause the overactivation of microglia in the cerebella of iPLA_2_β^-/-^ mice? It is known that the oxidation of mitochondrial phospholipids, particularly cardiolipin, sets cytochrome c free and release to trigger apoptosis [28]. Mitochondria are the primary source of ROS in the CNS [29] and the inner mitochondrial membranes are particularly susceptible to ROS attack because they contain a high proportion of cardiolipin, which is very rich in polyunsaturated fatty acids [30], [31]. Accumulating evidence suggests that iPLA_2_β-mediated deacylation of phospholipid is a critical step in maintaining optimal mitochondrial membrane composition by participating in cardiolipin remodeling and in repairing of oxidative modifications caused by mitochondrial ROS production [32], [33], [34], [35], [36], [37], [38], [39]. When iPLA_2_β is dysfunctional or missing, Purkinje cells in the cerebellum may be sensitized to apoptotic induction.

Microglia are phagocytes of myeloid origin that function as the principal immune effectors in the CNS [40]. They work to promptly clear apoptotic neuronal cells and prevent the secondary necrosis and inflammation that can further damage neighboring neural cells [41]. Previous studies suggest iPLA_2_β can be irreversibly activated by caspase-3 activation during apoptosis [1], [42], [43] and this activation produces the chemoattractant lysophosphatidylcholine to recruit professional phagocytes to engulf the apoptotic cells [43]. Effective clearance of apoptotic cells is an important physiologic homeostatic mechanism that is required to prevent secondary necrosis and inflammation [44], [45]. In the absence of iPLA_2_β, apoptotic Purkinje cells may not be promptly cleared, which can cause overactivation of microglia [24]. These overactive microglia can produce highly neurotoxic factors, including superoxide, TNF-α, and IL-1β, which are likely to perpetuate neuronal death and lead to overt cerebellar atrophy in iPLA_2_β^-/-^ mice.

In summary, Purkinje cell loss is a pathological characteristic of cerebellar atrophy of the iPLA_2_β^-/-^ mice. The loss of functional iPLA_2_β in vivo may result in the increase in apoptosis and the decrease in apoptotic cell clearance, which instigate inflammation. The presence of reactive astrogliosis, the activation of microglial cells, and the up-regulation of the proinflammatory cytokines TNF-α and IL-1β in the cerebella of the iPLA_2_β^-/-^ mice suggest that inflammation occurs and inflammation-mediated neurotoxicity may play a role in the pathogenesis of cerebellar atrophy in INAD. These findings suggest that in vivo microglial imaging by positron emission tomography (PET) may allow the assessment of disease progression [24] and that early intervention with anti-inflammatory therapy may help slow, if not prevent or reverse, the cerebellar atrophy in INAD.

## Materials and Methods

### Animals

iPLA_2_β^-/-^ mice (C57/BL6 background) and their wild type (WT) littermates were obtained by mating heterozygote iPLA_2_β^+/-^ mice. Mice were housed in a temperature- and humidity-controlled facility with a 12-hour light/dark cycle with standard chow diet and water *ad libitum*. All animal care and experimental procedures were approved by the Institutional Animal Care and Use Committee (IACUC) at the Mount Sinai School of Medicine.

### Gait stride test

Gait stride test was performed as described previously [46]. Briefly, mice in each group were given three training runs each day for three days, assisted by the home cage on the first day and unassisted on the next two days. On the fourth day (test), the mouse's paws were coated with ink (red ink on the hindpaws and blue on the forepaws), and paw prints were recorded on two consecutive runs. Records that displayed the clearest prints and the most consistent gait for five consecutive strides were chosen for analysis.

### Cerebellar measurements

iPLA_2_β^-/-^ mice and their age-matched WT littermates (n = 15/group) were deeply anesthetized by intraperitoneal injection of a mixture of 100 mg/kg of Ketamine and 20 mg/kg of Xylazine. Cerebella were carefully collected and weighted.

### Cerebellum preparation and immunohistochemistry

Mice were anesthetized by intraperitoneal injection of a mixture of 100 mg/kg of Ketamine and 20 mg/kg of Xylazine and perfused with 4% paraformaldehyde via puncture of the left ventricle. The cerebella were removed and further fixed in 4% paraformaldehyde at 4°C overnight, and then cryopreserved in 30% sucrose.

The cerebellum was cut into 20 µm sections by Leica Cryomicrotome (Wetzlar, Germany) and standard immunostaining was performed using following primary antibodies: rat anti-GFAP ( 1∶1000; Invitrogen, Carlsbad, CA), rabbit polyclonal anti-calbindin (1∶100; Abcam, Cambridge, MA), and rabbit polyclonal anti-Iba-1 (1∶1000; Biocompare, Richmond, VA). Goat anti-rat/rabbit IgG with the Alexa Fluor® dyes (Alexa Fluor® 488, Invitrogen, Carlsbad, CA) and DyLight® 594 (red) (Vector Laboratories, Burlingame, CA) were used for the secondary antibodies. The sections were mounted with VECTASHIELD® Hard Set™ Mounting Medium with DAPI (VECTOR Laboratories, Burlingame, CA) and coverslipped. All images were captured by a Zeiss Axioplan 2 microscope and a confocal microscope (Zeiss LSM 510 META).

### Purkinje cell and microglia counts

Purkinje cells in the cerebella were analyzed as described previously [47]. Briefly, Purkinje cells were quantified by stereologically counting calbindin positive-cell bodies in 10× images from six entire cerebellar sections of each animal (30 µm thick and 180 µm apart) were analyzed (control, *n* = 6; iPLA_2_β^-/-^, *n* = 8). Microglial cells in the molecular layer of the cerebellum were quantified by counting Iba-1-positive cells.

### Western Blot Analysis

Mice were decapitated and cerebella were dissected on ice and quickly immersed in the buffer (20 mM HEPES, pH 7.4/1 mM MgCl_2_/0.25 mM CaCl_2_/protease inhibitor cocktail/PMSF-pepstatin/phosphatase inhibitor/DNase 80 U/ml) and homogenized by Dounce Tissue Homogenizer (Eberbach). Protein was determined by the BCA method (Pierce), and aliquots of protein per sample were separated by SDS-PAGE, transferred to nitrocellulose, blotted with corresponding antibodies, and detected by ECL WESTERN blotting detection system (Amersham Biosciences). Anti- GFAP antibody (1∶1000, Invitrogen), anti-Iba-1 antibody (1∶1000, Biocompare), and anti-actin antibodies (Santa Cruz) were used for protein detection.

### Quantitative Real-time RT-PCR analysis

Total RNA was extracted from the cerebella collected from the iPLA_2_β^-/-^ and age-matched WT mice using an RNeasy Mini Kit. Primers and probes used in quantitative real-time RT-PCR (qRT-PCR) were designed by PrimerQuest^SM^ (IDT SciTools, Coralville, IA) as follows: GFAP, 5′-TCT GAA CCC TCT GAG CAA ATG CCT-3′ and 5′-ATT CAC TAC AAG AGC AGC CGT CCA-3′, 56-FAM/TG TGG GCA G/Zen/G CTC TGT GTT TGA TTC A/3IABkFQ; Iba-1, 5′-ATG AGC CAA AGC AGG GAT TTG CAG-3′ and 5′-AGT TTG GAC GGC AGA TCC TCA TCA-3′, 56-FAM/TT TGG ACT G/Zen/C TGA AGG CCC AGC AGG AA/3IABkFQ; TNF-α, 5′-TCTCATGCACCAC CATCAAGACT-3′ and 5′-ACCACTCTCCCTTTGCAGAACTCA-3′, 56-FAM/AATGGGCTT/Zen/TCCGAATTCA CTGGACCT/3IABkFQ; IL-1β, 5′-AAGGGCTGCTTCC AAACCTTTGAC-3′ and 5′-ATACTGCCTGCCTGAAGCTCTTGT-3′, 56-FAM/ATC CAGCT T/Zen/CAAATCTCG CAGCAGCA/3IABkFQ; GAPDH, 5′-TCAACAGCAAC TCCCACTCTTCCA-3′ and 5′-ACCCTGTTGCTGTAGCCGTATTCA, 56-FAM/GGCT GGCAT/Zen/TGCTCTCAATGAC AACT/3IABkFQ. qRT-PCR was performed using an EXPRESS One-Step SuperScript qRT-PCR Kit from Invitrogen (Carlsbad, CA) according to the manufacturer's instructions on *7900HT Real*-*Time* PCR Systems (Applied Biosystems, Foster City, CA). The mRNA levels were calculated by comparative CT methods (X_Test_/X_GAPDH_ = 2^ΔΔC^_T_) with GAPDH as the endogenous reference gene.

### IL-1β and TNF-α measurements

The levels of cytokines in cerebellum homogenates were measured as described previously with a modification [48]. Briefly, mouse blood was collected before sacrificing. The mice were anesthetized by intraperitoneal injection of a mixture of 100 mg/kg of Ketamine and 20 mg/kg of Xylazine. Then cerebella were removed, snap frozen in liquid nitrogen, and stored at −80°C until use. Cerebellar samples were placed in sterile PBS containing protease inhibitor cocktail (300 µl solution per 10 mg), homogenized using Tissuemiser (Fisher Scientific, Pittsburgh, PA), and centrifuged at 11,000 rpm at 4°C for 20 minutes. The supernatants or blood were aliquoted and cytokine levels were determined with mouse TNF-α and IL-1β ELISA Kits (eBioscience, Inc., San Diago, CA). Cytokine levels were normalized with total proteins and expressed as ng/mg proteins.

### Statistics

Data were expressed as means ± SEM. Significant differences were evaluated by unpaired two-tailed Student's *t* test by using PRISM. Significance levels are described in individual figure legends.

## Supporting Information

Figure S1 **The nissle staining analysis of mouse cerebellum.** Nissle staining was performed according to standard methods. Briefly, the sagittal sections of cerebella from 13-month-old iPLA_2_β^-/-^ mice and the age-matched wild type littermates (WT) were mounted on slides, dehydrated and rehydrated in graded ethanols and xylenes, incubated in 1% cresyl violet for 30 sec, decolorized in acetic acid, and then dehydrated and coverslipped and analyzed by a a Zeiss Axioplan 2 microscope. The parasagittal views of the mouse cerebella of the nissl staining revealed no apparent anatomical differences between the iPLA_2_β^-/-^ and their wild type littermates with the exception of the less intensive staining in the granule cell layer of cerebella in iPLA_2_β^-/-^ mice. ml, molecular layer; gcl, granule cell layer; DCN, deep cerebellar nucleus. Scale bar, 100 µm.
(TIF)

Figure S2 **The Calbindin staining analysis of mouse cerebellum.** The cerebellum was cut into 20 µm sections by Leica Cryomicrotome (Wetzlar, Germany) and standard immunostaining was performed by rat anti-GFAP ( 1:1000; Invitrogen, Carlsbad, CA), rabbit polyclonal anti-calbindin-2 (1:100; abcam, Cambridge, MA), Goat anti-rat IgG with the Alexa Fluor® dyes (Alexa Fluor® 488, Invitrogen, Carlsbad, CA) were used for the secondary antibodies. Calbindin staining (green) showed the Purkinje cell lose and degeneration of PC dendrites in iPLA_2_β^-/-^ mice cerebella. ml, molecular layer; gcl, granule cell layer; DCN, deep cerebellar nucleus. Scale bar, 100 µm.
(TIF)
