# Supplementary figures and images for: Genetic Ablation of PLA2G6 in Mice Leads to Cerebellar Atrophy Characterized by Purkinje Cell Loss and Glial Cell Activation
Source: PLoS One. 2011 Oct 28;6(10):e26991. doi: 10.1371/journal.pone.0026991 (PMC3203935; doi:10.1371/journal.pone.0026991)

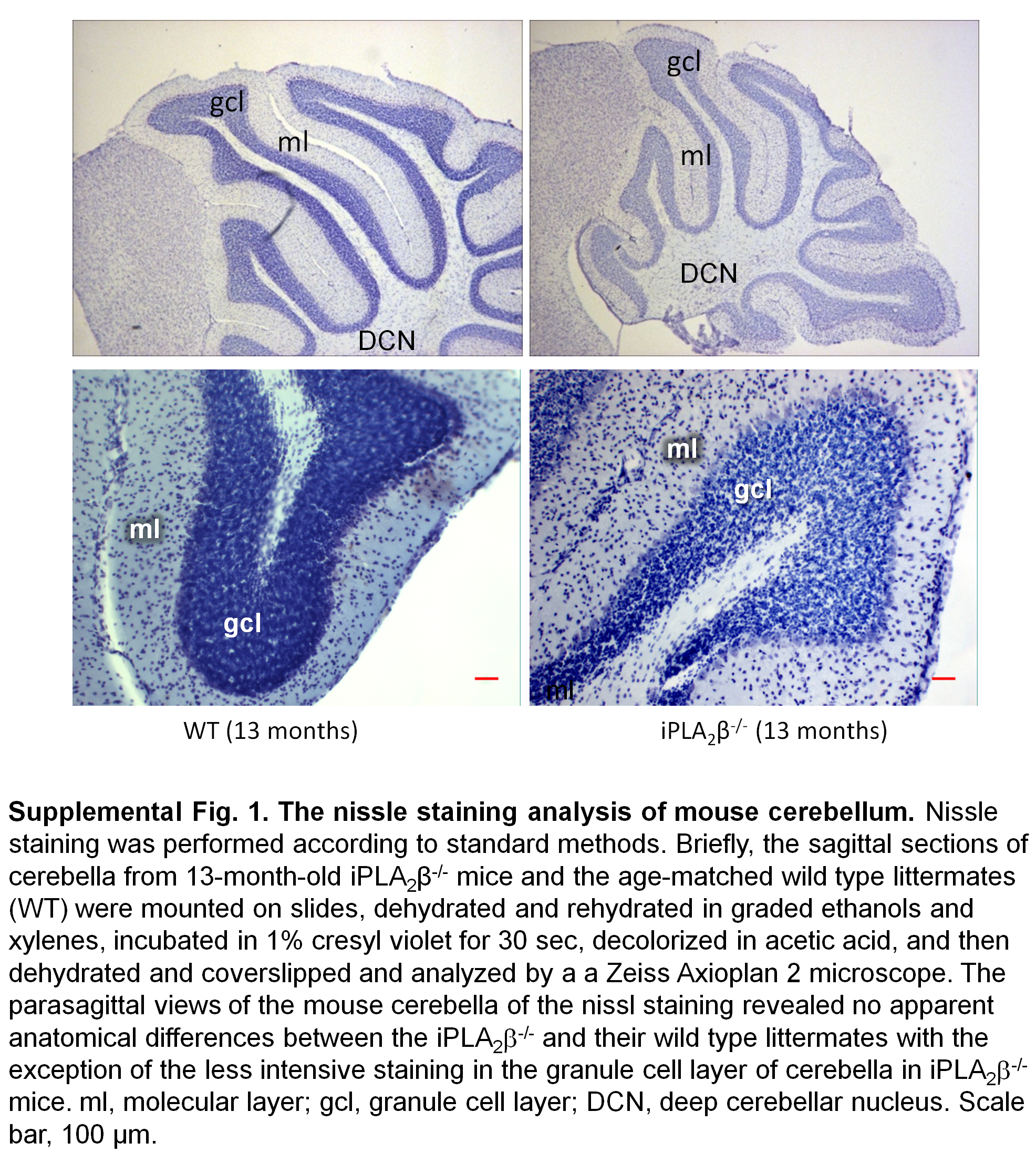

Supplement: Figure S1 — The nissle staining analysis of mouse cerebellum. Nissle staining was performed according to standard methods. Briefly, the sagittal sections of cerebella from 13-month-old iPLA2β-/- mice and the age-matched wild type littermates (WT) were mounted on slides, dehydrated and rehydrated in graded ethanols and xylenes, incubated in 1% cresyl violet for 30 sec, decolorized in acetic acid, and then dehydrated and coverslipped and analyzed by a a Zeiss Axioplan 2 microscope. The parasagittal views of the mouse cerebella of the nissl staining revealed no apparent anatomical differences between the iPLA2β-/- and their wild type littermates with the exception of the less intensive staining in the granule cell layer of cerebella in iPLA2β-/- mice. ml, molecular layer; gcl, granule cell layer; DCN, deep cerebellar nucleus. Scale bar, 100 µm. (TIF) [file pone.0026991.s001.tif]

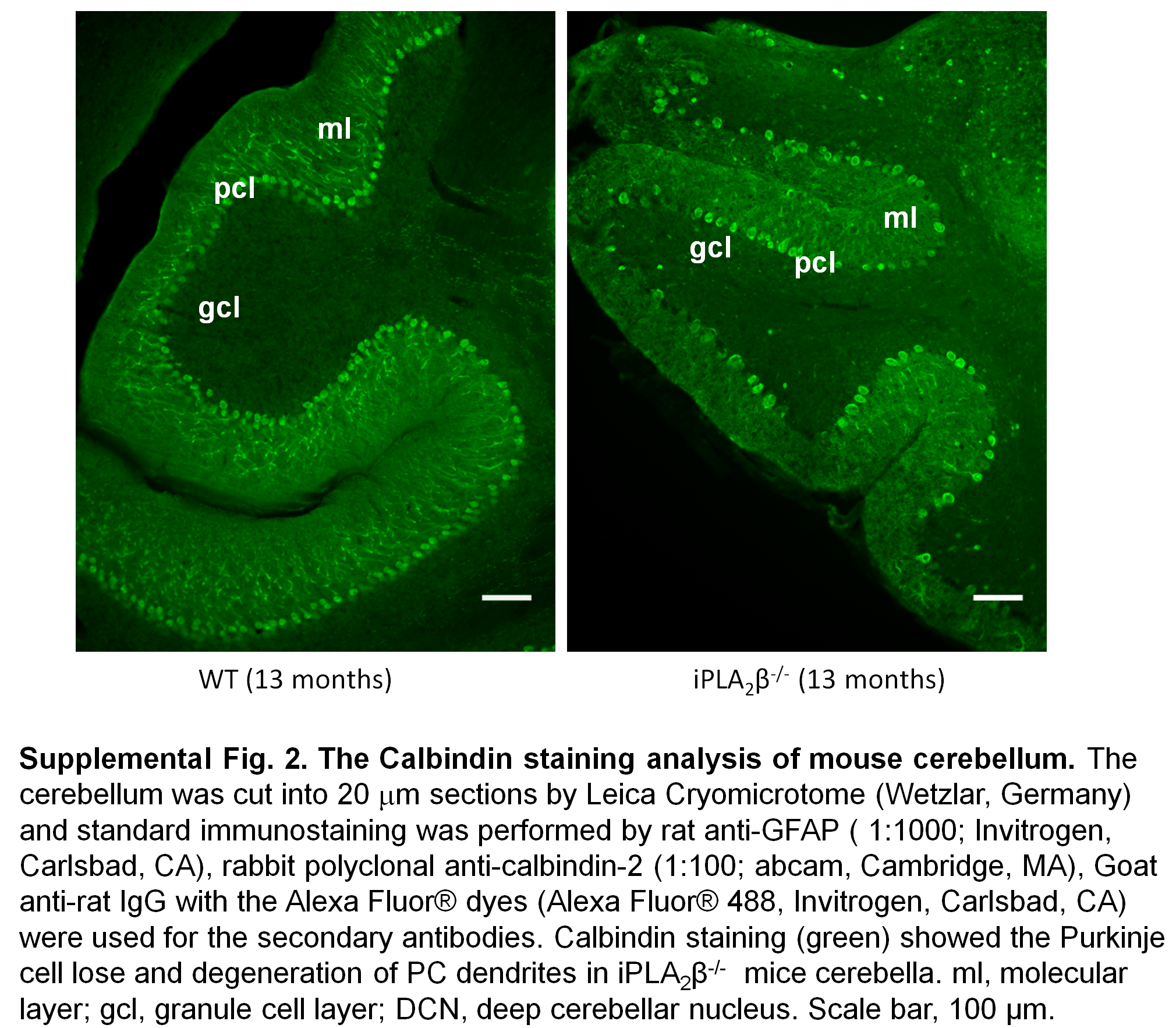

Supplement: Figure S2 — The Calbindin staining analysis of mouse cerebellum. The cerebellum was cut into 20 µm sections by Leica Cryomicrotome (Wetzlar, Germany) and standard immunostaining was performed by rat anti-GFAP ( 1:1000; Invitrogen, Carlsbad, CA), rabbit polyclonal anti-calbindin-2 (1:100; abcam, Cambridge, MA), Goat anti-rat IgG with the Alexa Fluor® dyes (Alexa Fluor® 488, Invitrogen, Carlsbad, CA) were used for the secondary antibodies. Calbindin staining (green) showed the Purkinje cell lose and degeneration of PC dendrites in iPLA2β-/- mice cerebella. ml, molecular layer; gcl, granule cell layer; DCN, deep cerebellar nucleus. Scale bar, 100 µm. (TIF) [file pone.0026991.s002.tif]
